# Psychometric Properties of the Persian Version of the Youth Risk Behavior Survey Questionnaire

**Published:** 2012-06-30

**Authors:** A Baheiraei, Z Hamzehgardeshi, M R Mohammadi, S Nedjat, E Mohammadi

**Affiliations:** 1 Department of Reproductive Health, Tehran University of Medical Sciences, Tehran, Iran; 2 Department of Midwifery, Mazandaran University of Medical Sciences, Sari, Iran; 3 Department of Psychiatric, Psychiatry and Psychology Research Centre, Tehran University of Medical Sciences, Tehran, Iran; 4 School of Public Health, Knowledge Utilization Research Center, Tehran University of Medical Sciences, Tehran, Iran; 5 Department of Nursing, Tarbiat Modares University, Tehran, Iran

**Keywords:** Risk behaviour, Youth, Persian version, Psychometric properties, Survey questionnaire

## Abstract

**Background:**

Adolescents may get involved in high-risk behaviors. Surveys are the primary, and sometimes the sole source of data collection for many high-risk health behaviours. We examined the reliability and validity of the psychometric properties of the self-administered Persian version of the 2009 Youth Risk Behavior Surveillance System (YRBSS) questionnaire.

**Methods:**

In a methodological study in summer 2010, 100 Iranian adolescents aged 15-18 years were recruited through convenience sampling. The face and content validity were used for the questionnaire validity. In order to evaluate the questionnaire’s reliability, the Intraclass Correlation Coefficient (ICC) and Cronbach’s α were calculated for domains and 89 items.

**Results:**

Among 89 items, the ICC values were below 0.4 (weak reliability) for 2 items (2.25%), 0.4-0.6 (moderate reliability) for 10 items (11.24%), 0.6-0.8 (good reliability) for 32 items (35.96%) and 0.8-1 (excellent reliability) for 45 items (50.56%). The prevalence of most high-risk behaviors was constant in the first and second survey. The value of Cronbach’s α was 0.73 for intentional and unintentional injuries, 0.77 for tobacco use, 0.86 for alcohol and other drug use, and 0.79 for unsafe sexual behaviors. No domain had a mean ICC of below 0.6. Furthermore, 97.75% of the items had moderate to excellent reliability. Thus, the Persian YRBSS questionnaire had an acceptable reliability.

**Conclusion:**

Over the 2-week period, sexual behaviors were reported with less consistency compared to other behaviors. In any case, researchers must be aware of the limitation of the data collected through this questionnaire, particularly in comparison to the domain of sexual behaviors. Overall, 97.75% of the items had moderate to excellent reliability. Thus, the Persian YRBSS questionnaire had an acceptable reliability.

## Introduction

Adolescents may get involved in high-risk behaviors such as smoking, drug abuse and early and unprotected sexual activity which will jeopardize their health in the future. Surveys are the primary, and sometimes the sole source of data collection for many high-risk health behaviours.[1] The Youth Risk Behavior System Surveillance (YRBSS) is systematic epidemiologic surveillance system prepared by the Center for Disease Control and Prevention (CDC) for the purpose of monitoring youth risk behaviors. It focuses on risk behaviors that develop during youth and will result in mortality, morbidity, complications and behavioral problems in youth and adulthood. These behaviors include unintentional and intentional injuries, tobacco use, alcohol and other drug use, high-risk sexual behaviors that lead to HIV infection, other sexually transmitted diseases (STDs) and unintended pregnancies, dietary behaviors, physical activity as well as weight gain and asthma.[2][3]

The first version of the YRBSS questionnaire was prepared by the CDC for the first national American survey. The surveys were conducted biannually and the questionnaires were revised prior to each survey, if required. In 1997, CDC determined the goals of health people in 2010 and thus required the YRBSS plan to warrant the information. Therefore, some changes were made to the YRBSS in 1999.[4] Finally, minor revisions were made to the questionnaire in 2001, 2003, 2005, 2007 and 2009.

It is crucial to evaluate the reliability of the survey data in order to determine the accuracy of the measurement scales over time as well as to determine the prevalence of high-risk behaviors to identify target groups in public health interventions. Inaccurate data may easily lead to errors in policy making and evaluation of interventions.[5] Reliability studies of CDC’s youth risk behaviors have indicated a high reliability for a wide range of self-reported risk behaviors.[4][5][6][7][8]

No systematic surveillance program for adolescence risk behaviors exists in Iran. Numerous studies have been conducted to deal with one or more of the adolescent risk behaviors such as drug abuse,[9][10] tobacco use,[11][12] intentional and unintentional injuries,[13] reproductive health behaviors,[14] high-risk sexual behaviors in boys,[15] obesity[16] and suicide.[17] However, only a few studies have dealt with comprehensive assessment of high-risk behaviors using the YRBSS,[18][19] and they were not based on adolescence populations. Since the findings of these surveys are helpful for policy making in the field of youth health in local, national and international levels, it is essential to ensure the accuracy of self-reports for questions related to high-risk behaviors. For this purpose, many studies have been conducted worldwide to address the reliability of the YRBSS questionnaire.[2][4][20] A review of these studies indicates that the YRBS questionnaire has an acceptable reliability. Nevertheless, due to the self-administered nature of the questionnaire, the standardized English version of the questionnaire is not applicable in Iran. In addition, the cultural and demographic differences warrant validity of the YRBSS tool. Since the reliability of the YRBS questionnaire has not been studied in Iranian youth population, the current study assesses the reliability and validity of the psychometric indices of the Persian version of the self-administered 2009 YRBSS questionnaire, as well as presenting the first evaluation of the reliability and validity of the Persian YRBSS questionnaire.

## Materials and Methods

The present study is a methodological study for the purpose of tool validity and reliability. In summer 2010, 100 Iranian adolescents aged 15-18 years willing to participate in this survey were recruited through convenience sampling. In the first and second surveys, 98 adolescents (53 boys and 45 girls) thoroughly completed the questionnaires. Two adolescents, who had not thoroughly completed the questionnaires in the two surveys, were excluded from the analysis. The responding rate for the questionnaires was 98%.

The state and national YRBS 2009 questionnaire is a standardized self-administered questionnaire consisting of 87 items. The Persian version of this questionnaire (PYRBS) is a self-administered questionnaire adapted from the 2009 YRBS. Furthermore, a few items have been added to cover intentional injuries and water pipe use, and one item pertaining to asthma has been eliminated. The PYRBS questionnaire consists of 89 items.

Initially, the YRBSS questionnaire was separately translated by two Persian translators. It was then discussed by translators and proficient professors of health sciences to omit certain problems. The version was subsequently edited and commissioned to an English professor to be translated back into English. The purpose was to ensure the accuracy of the translation. Subsequently, the translated (English) and original versions of the questionnaire were compared and contrasted. The final questionnaire was submitted to eight professors in the fields of health, psychology and psychiatry of Tehran University of Medical Sciences, Mazandaran University of Medical Sciences and Tarbiat-Modarres University of Medical Sciences. Due to the relatively low prevalence of self-reporting statistics for asthma in Iran,[21][22] the asthma related questions were removed. Moreover, due to the increased prevalence of water pipe smoking in many parts of the world, especially in the Eastern Mediterranean region,[23] the questions on water pipe smoking were added to the smoking questions.

The pilot study was conducted on 10 adolescents, resulting in minor changes in the questionnaire. The objective of this stage was to determine whether or not the adolescents’ inference from the statements of the questionnaire was compatible with the implications and purpose pursued by the researcher, as well as to determine whether or not the adolescents had similar inferences from one statement. For this purpose, after the adolescents completed the questionnaires individually, they were inquired about the questions and a discussion and evaluation were conducted after the completion of the task. Subsequently, challenges resulting from the first preliminary study were discussed by the research group and necessary amendments were made. A secondary preliminary study was conducted using the same method on 20 adolescents in order to confirm the accuracy of the translation. Based on the findings of the second preliminary study, minor changes were made on the wording used in three questions. The final question-naire was submitted to four professors for a final revision. Ultimately, the face validity and content validity were used for the questionnaire validity.

The Ethics Committee of Tehran University of Medical Sciences (code number: 89-01-28-10494) approved the data collection protocol of the study. Consistent with previous studies, test re-test was used. Two series of anonymous questionnaires were attributed a code from 1-100. Each adolescent received a code and was advised to memorize it. Each envelope contained two coded questionnaires. Adolescents and their parents expressed their informed consent orally for participation in the study. On the first referral of data collectors to the houses, the first questionnaire was completed by the adolescents. Subsequently, the data collectors reminded them that they would return two weeks later to collect the second questionnaire, also completed by the adolescents. In order to ensure the confidentiality of questionnaires, they were collected in a box. Moreover, the data collectors reassured the adolescents that their information would remain confidential.

To assess the reliability of the test re-test, 98 adolescents aged 15-18 years who had completed the two YRBSS questionnaires were enrolled. In order to evaluate the questionnaire’s reliability, the Intraclass Correlation Coefficient (ICC) was calculated for all domains and items. Furthermore, Cronbach’s α was calculated to evaluate the questionnaire’s reliability in the dimension of internal consistency. In addition, the frequency of behaviors in the first and second survey was calculated for all items. The following criteria were used as quantitative values for ICC: below 0.4 (weak reliability), 04.-0.6 (moderate reliability), 0.6-0.8 (good reliability) and 0.8-1 (excellent reliability).[24] In the present study, questions with ICC values of less than 0.6 were revised. Values of Cronbach’s α above 0.7 were described as good internal consistency. Data were analyzed using Statistical Package for Social Sciences (Version 16 for windows, SPSS Inc., Chicago, USA) and STATA version 10.

## Results

The 98 adolescents aged 15-18 years consisted of 51 (52%) girls and 47 (48%) boys. The mean age of the participants was 16.34 years (SD=1.66).

The estimated reliability for 89 items of the IYRBS questionnaire and the rate of prevalence of high-risk behaviors of the first and second survey are summarized in Table 1. Among these items, the ICC values were below 0.4 (weak reliability) for 2 items (2.25%), 0.4-0.6 (moderate reliability) for 10 items (11.24%), 0.6-0.8 (good reliability) for 32 items (35.96%) and 0.8-1 (excellent reliability) for 45 items (50.56%). The prevalence of most high-risk behaviors was constant in the first and second survey. Out of the 19 questions in the domain of intentional and unintentional injuries, only exposure to bullying by others had an ICC value of below 0.6 (0.54).

**Table 1:** Reliability index in the dimension of repeatability of questionnaire items and prevalence of high-risk behaviors on first and second surveys.

**** **Items**	**ICC^a^**			**Prevalence (%)**	
	**Mean**	**Minimum**	**Maximum**	**Time 1**	**Time 2**
Wearing a helmet rarely or never on a motor bike	0.91	0.87	0.94	53.1	51
Fastening seatbelt rarely or never when in a car	0.88	0.82	0.92	20.4	19.3
Riding a car with an intoxicated driver during the last 30 days	0.91	0.87	0.94	46.9	46.9
Driving under intoxication	0.93	0.89	0.95	11.2	12.2
Driving without a license during the last 30 days	0.95	0.92	0.96	48.9	50
Racing or swerving with a motor vehicle during the last 30 days	0.97	0.96	0.98	27.8	34.7
Carrying a cold weapon during the last 30 days	0.95	0.93	0.97	46.3	48.5
Staying at home because of feeling insecure during the last 30 days	0.68	0.53	0.79	15.3	27.6
Being threatened or beaten with cold weapon during the last 30 days	0.84	0.76	0.89	18.4	20.4
Taking part in a physical fight during the last 12 months	0.92	0.88	0.94	45.9	46.9
Being injured in physical fight and referring to a healthcare center during the last 12 months	0.76	0.64	0.83	10.2	12.2
Stealing money from parents during the last 12 months	0.94	0.92	0.96	36.7	37.9
Being hit, slap, or physically hurt by a boyfriend or girlfriend during the last 12 months	0.77	0.66	0.85	11.3	9.2
Being physically forced into sexual relationship	0.83	0.75	0.88	13.3	9.2
Exposure to bullying by others during the last 12 months	0.54	0.32	0.69	24.4	15.3
Feeling despair and sadness during the last 12 months	0.72	0.59	0.81	48	51
Having suicidal thoughts during the last 12 months	0.83	0.74	0.88	27.6	25.5
Having a serious plan for suicide during the last 12 months	0.87	0.81	0.91	25.5	21.4
Trying suicide during the last 12 months	0.74	0.61	0.82	8.2	8.2
Having experienced smoking cigarettes even as little as one two puffs	0.74	0.61	0.82	45.9	41.8
Experiencing the first cigarette under 13 years of age	0.78	0.67	0.85	63.7	68.8
Smoking cigarettes ≥1 day during the last 30 days	0.89	0.83	0.92	33	30.8
Smoking ≥ 1 cigarette during the last 30 days	0.68	0.53	0.79	20.6	18.4
Buying cigarettes from a shop or supermarket during the last 30 days	0.89	0.83	0.92	9.2	6.2
Smoking cigarettes on a daily basis during the last 30 days	0.27	0.81	0.51	16.3	14.4
Trying to quit smoking during the last 12 months	0.87	0.81	0.91	23.5	20.4
Having experienced hookah even as little as one or two puffs	0.56	0.33	0.70	73.2	60.3
Using a communal water pipe	0.63	0.43	0.74	30.9	28.9
Using water pipe ≥ 1 day during the last 30 days	0.92	0.88	0.94	53.1	50.4
Smoking by family members	0.72	0.58	0.81	47.4	51
water pipe use by family members	0.85	0.78	0.90	56.1	58.2
Having experiences alcoholic beverages even as little as one or two sips	0.60	0.40	0.73	30.2	20.3
Drinking alcohol ≥ 1 day throughout the entire life	0.78	0.67	0.85	40.2	30.6
Drinking the first alcoholic drink below 13 years of age	0.71	0.56	0.80	70.8	74.4
Drinking alcohol ≥1 day during the last 30 days	0.85	0.79	0.90	28.6	24.4
Drinking alcohol 5 times or more in ≥ 1 day during the last 30 days	0.93	0.89	0.95	12.2	10.2
Drinking alcohol in parties with friends	0.78	0.68	0.85	19.8	16.3
Drinking alcohol with friends	0.82	0.75	0.89	90.6	89.7
Having experienced narcotics	0.67	0.51	0.78	7.1	5.1
Using narcotics in parties with friends	0.72	0.59	0.81	17	15.1
Using narcotics ≥1 day	0.52	0.28	0.68	2.7	3.3
Using the first narcotic below 13 years of age	0.72	0.59	0.81	78.2	78.3
Using weed and opium ≥1 day during the last 30 days	0.51	0.27	0.67	4.4	3.2
Having experienced using glass and crack	0.88	0.83	0.92	6.2	7
Using glass and crack ≥1 day during the last 30 days	0.89	0.84	0.93	4.1	3.1
Having experienced heroin	0.54	0.28	0.72	2.7	3.1
Having experienced methamphetamines like crystal and ice	0.69	0.54	0.79	5.1	3.1
Having experienced using Ecstasy pills	0.68	0.52	0.78	8.2	6.4
Having used steroid tablets without prescription and for bodybuilding	0.95	0.93	0.97	11.2	11.2
Having used Ritalin without prescription	0.81	0.72	0.87	2.1	1.1
Having injected illegal drugs	0.63	0.45	0.75	1.1	1.3
Receiving suggestions for narcotic use from others	0.70	0.55	0.79	52	49.9
Having smoker friends	0.46	0.19	0.63	73.2	72.2
Having drinker friends	0.74	0.61	0.82	64.3	61.2
Having friends who use narcotics	0.75	0.62	0.83	15.5	14.1
Having friends who use psychedelics	0.61	0.42	0.74	50	49.9
Having experienced a boyfriend/girlfriend	0.79	0.69	0.86	66	66.7
Contact with boyfriend/girlfriend at home	0.81	0.72	0.87	35.7	34.6
Having experienced sexual contact	0.45	0.18	0.63	18.8	19.7
First sexual contact below 13 years of age	0.72	0.58	0.81	56.7	54.7
Having had sexual contact with ≥4 partners throughout the entire life	0.81	0.72	0.87	9.2	6.1
Using alcoholic drinks or other drugs before latest sexual intercourse	-0.01	0.51	0.32	14.6	15.7
Not using condom on the latest sexual contact	0.92	0.88	0.94	4.1	3.1
Not using contraceptive methods on the latest sexual contact	0.70	0.55	0.80	59.2	60.6
Describing self as fat	0.88	0.82	0.92	18.4	17.7
Trying to lose weight	0.82	0.73	0.88	33	30.6
Exercising for weight loss or fixation during the last 30 days	0.75	0.63	0.83	31.2	29.1
Using low-fat and low-calorie food for weight loss or fixation during the last 30 days	0.83	0.85	0.88	26.5	27.6
Fasting ≥24 hours for weight loss or fixation during the last 30 days	0.97	0.95	0.98	10.2	9.2
Using diet tablets and powders for weight loss or fixation during the last 30 days	0.97	0.96	0.98	13.3	12.2
Vomiting or using laxatives for weight loss or fixation during the last 30 days	0.96	0.95	0.97	8.2	7.1
Drinking fruit juice ≥2 times per day during the last 7 days	0.87	0.80	0.91	3.1	2.1
Eating vegetable salad ≥3 times per day during the last 7 days	0.86	0.80	0.91	1	0
Eating vegetables ≥ 3 times per day during the last 7 days	0.71	0.58	0.81	0	1.1
Consuming gaseous drinks and canned food ≥1 time per day during the last 7 days	0.81	0.72	0.87	13.1	14.2
Consuming milk and dairy products ≥3 times per day during the last 7 days	0.87	0.81	0.91	2.1	2.1
Exercising or having a physical activity ≥20 minutes, ≥3 days during the last 7 days, with the intensity to induce perspiration and quickening of breath	0.91	0.86	0.94	24.5	22.2
Exercising or having a physical activity ≥20 minutes, ≥5 days during the last 7 days, without perspiration or quickening of breath	0.87	0.81	0.91	5.1	5.1
Exercising or having a physical activity ≥60 minutes, ≥1 day during the last 7 days	0.89	0.83	0.92	1	0
Watching TV ≥3 hours per day on weekdays	0.82	0.74	0.88	42.9	42.5
Playing video games ≥3 hours per day on weekdays	0.91	0.87	0.94	80.6	83.7
Participating in sports at school ≥1 day during the last 7 days	0.64	0.46	0.76	91.8	80.6
Exercising more than 20 minutes on school’s physical education class	0.82	0.74	0.88	34.7	22.4
Membership in ≥1 sports team during the last 12 months	0.77	0.66	0.84	85.7	84.4
Being educated about AIDS at school	0.61	0.42	0.74	30.9	28.9
Taking the HIV test	0.47	0.22	0.65	19.4	16.3
Using sun-blocks rarely or never when leaving home for more than 1 hour	0.95	0.92	0.96	19.4	18.3
Asking counsel from an adult, teacher or adviser during the last 12 months	0.43	0.16	0.62	2.3	1.1
Sleeping ≥7 hours at night on weekdays	0.54	0.32	0.69	78.7	69.9

^a^ Intra-class Correlation Coefficient, Bold: Mean ICC below 0.6

In the domain of tobacco use, 2 out of 12 questions had ICC values of below 0.6. Smoking at least one cigarette per day over 30 days and experiencing water pipe even as little as one or two puffs achieved ICC scores of 0.27 and 0.56, respectively.

In the domain of alcohol and other drug use, 4 out of 25 questions had ICC values of below 0.6. Frequency of drug abuse in life, frequency of opium and marijuana use in the last 30 days, frequency of heroin use, and having smoking friends achieved ICC scores of 0.52, 0.51, 0.54 and 0.46, respectively.

Out of the 8 questions in the domain of unsafe sexual behaviors, experiencing sex with the opposite sex and using alcohol or drugs before the last sex scored 0.45 and - 0.01, respectively. No question in the domain of physical activity and body weight and dietary behaviors had ICC score of below 0.6.

Out of the 5 questions related to other health-related issues, history of HIV test, history of asking counsel from a teacher, advisor or adult about a personal problem, and sleeping hours on weekdays received ICC scores of below 0.6. Table 2 demonstrates the mean ICC for domains of the YRBSS questionnaire. The mean values of ICC for domains of intentional and unintentional injuries, tobacco use, alcohol and other drug use, high-risk sexual behaviors, body weight and dietary behaviors, physical activity and other health-related issues were 0.83, 0.73, 0.71, 0.64, 0.85, 0.82 and 0.60, respectively.

**Table 2:** Mean reliability index in the dimension of repeatability of questionnaire items for domains of risk behaviours.

**Domains**	**ICC^a^**			
	**Questions (n)**	**Mean**	**Minimum**	**Maximum**
Unintentional and intentional injuries	19	0.83	0.54	0.97
Tobacco use	12	0.73	0.27	0.92
Alcohol and other drug use	25	0.71	0.46	0.95
High-risk sexual behaviors	8	0.64	- 0.01	0.92
Body weight and dietary behaviors	12	0.85	0.71	0.97
Physical activity	7	0.82	0.64	0.91
Other health-related issues	5	0.60	0.43	0.95

^a^ Intraclass Correlation Coefficient

Considering the diverse nature of the questions and the small number of questions in the domains, the value of Cronbach’s α was not calculable for all the domains. The value of Cronbach’s α was 0.73 in the domain of intentional and unintentional injuries, 0.77 in the domain of tobacco use, 0.86 in the domain of alcohol and other drug use, and 0.79 in the domain of unsafe sexual behaviors.

## Discussion

In the present study, the adolescents’ reports over a 2-week period were more reliable in the domains of intentional and unintentional injuries, body weight and dietary behaviors and physical activity compared to other domains of risk behaviors. Most items of intentional and unintentional injuries, tobacco use, alcohol use, substance abuse, nutritional behaviors and physical activity had relatively constant frequencies. In the short term, these behaviors may be reported constant, because few adolescents modify their behavior over a short period of two weeks. Over longer durations, these items may be reported with less consistency since adolescents report their status of sexual activity and substance abuse based on their current ideas[25][26][27] and thus, those adolescents who change their ideas and behaviors will result in more fluctuating reports.[26][27][28]

In evaluation of test re-test reliability, inconsistent responses on first and second tests may be interpreted as response errors. Such discrepancies may reflect an actual change. Considering this fact, estimates based on the findings of the study must be made with caution.[29] In order to ensure the accuracy of the data, not only the reliability, but also the validity of the data resulting from self-reporting muse be considered. Factors which may influence the validity and reliability of a survey include context of questions and the type of responses required from a cognitive point of view.[25] Confidentiality and anonymity also exert an appreciable impact on response error and increase the adolescents’ confidence. Furthermore, previous studies indicated that the rate of high-risk behaviors, such as use of tobacco, alcohol and other drugs, tended to be reported higher when using self-administered compared to interviewer-administered questionnaires.[30] One strong point of the present study was the use of self-administered questionnaires for adolescents. Nevertheless, underreporting is a possible drawback with this method.

The present findings indicate a high reliability for nutritional behaviors, which may be due to the consumption of home-made food. Thus, our adolescents did not modify their nutritional pattern in the 2 weeks. The findings of the present study indicate that the reliability of items pertaining to sexual behaviors is particularly critical. Cultural and legal obstacles make it quite difficult for adolescents to answer such questions. Due to the modification of present behaviors, adolescents change their report of past behaviors.[25][26][27]

In this study, almost 11.24% of YRBSS items scored average and 86.52% scored good or excellent for ICC. The items pertaining to alcohol or drug use prior to intercourse had a weak ICC. These items were not reliable, because adolescents who had reported using alcohol or drugs prior to sex on the first survey, did not report it on the second. A study with a larger sample size will circumvent this problem. It must be noted, however, that due to the unethical, illegal and criminal nature of these behaviors in the Iranian society, these questions prove particularly difficult for respondents to answer.

Consistent with the present findings, Zulling et al. (2006) reported a mean Kappa value of 0.69 for the domain of intentional and unintentional injuries, 0.70 for the domain of tobacco use, 0.43 for the domain of alcohol and other drug use, 0.70 for the domain of nutritional behaviors, 0.72 for the domain of physical activity and 0.60 for the domain of other health-related issues.[2] A review of previous studies indicated that questions with average or weak reliability were found in other cultures as well. In this study, no domain had a mean ICC of below 0.6. Furthermore, 97.75% of items had moderate to excellent reliability. Therefore, the calculated reliability is acceptable for all domains, with ICC values of 0.83 for the domain of intentional and unintentional injuries, 0.73 for tobacco use, 0.71 for alcohol and other drugs use, 0.64 for unsafe sexual behaviors, 0.85 for nutritional behaviors, 0.82 for physical activity and 0.60 for other health-related issues.

Over the 2-week period, adolescents consistently reported behaviors related to intentional and unintentional injuries, tobacco use, alcohol and other drug use, nutritional behaviors, physical activity and other health-related issues. However, sexual behaviors were reported with less consistency compared to other behaviors. This inconsistency is particularly problematic since adolescent sexual health is a significant aspect of public health and is of vital importance for the youth. Future revision of the YRBSS to envisage implicit questions about the adolescents’ latest sexual contacts are recommended. Researchers must be aware of the limitation of data collected through this questionnaire, particularly in comparison with the domain of sexual behaviors. Future studies on larger populations are required.
